# Molecular characterization of matrix metalloproteinase gene family across primates

**DOI:** 10.18632/aging.204021

**Published:** 2022-04-20

**Authors:** Yinglian Pan, Yadan Fan, Yanda Lu, Siyuan Peng, Haixue Lin, Qingchun Deng

**Affiliations:** 1 Department of Medical Oncology, The First Affiliated Hospital of Hainan Medical University, Haikou 570102, Hainan, People’s Republic of China; 2 Department of Gynecology, The Second Affiliated Hospital of Hainan Medical University, Haikou 570311, Hainan, People’s Republic of China

**Keywords:** matrix metalloproteinase, primate, gene structure, genomic organization, co-expression

## Abstract

Deregulation of matrix metalloproteinases (MMPs) contributes considerably to cancers, psychiatric disorders, macular degeneration and bone diseases. The use of humans in the development of MMPs as prognostic biomarkers and therapeutic targets is complicated by many factors, while primate models can be useful alternatives for this purpose. Here, we performed genome-enabled identification of putative MMPs across primate species, and comprehensively investigated the genes. Phylogenetic topology of the MMP family showed each type formulates a distinct clade, and was further clustered to classes, largely agreeing with classification based on biochemical properties and domain organization. Across primates, the excess of candidate sites of positive selection was detected for MMP-19, in addition to 1-3 sites in MMP-8, MMP-10 and MMP-26. MMP-26 showed *Ka/Ks* value above 1 between human and chimpanzee copies. We observed two copies of MMP-19 in the old-world monkey genomes, suggesting gene duplication at the early stage of or prior to the emergence of the lineage. Furin-activatable MMPs demonstrate the most variable properties regarding Domain organization and gene structure. During human aging, MMP-11 showed gradually decreased expression in testis, so as MMP-2, MMP-14, MMP15 and MMP-28 in ovary, while MMP-7 and MMP-21 showed elevated expression, implying their distinct roles in different reproductive organs. Co-expression clusters were formed among human MMPs both within and across classes, and expression correlation was observed in MMP genes across primates. Our results illuminate the utilization of MMPs for the discovery of prognostic biomarkers and therapeutic targets for aging-related diseases and carry new messages on MMP classification.

## INTRODUCTION

Matrix metalloproteinases (MMPs), also called matrixins, comprise a family of zinc-dependent endoproteinase. Collectively they are capable of degrading all extracellular matrix components (ECM) as well as non-matrix proteins [1]. The proteolytic activities of MMPs influence many fundamental physiological events involving tissue remodeling, embryogenesis, morphogenesis, angiogenesis, wound healing, and apoptosis [2–4]. Under pathological conditions, deregulation of MMP activity causes a variety of pathological outcomes including matrix weakening, tissue destruction, and fibrosis [2, 5].

Over recent years, aging-related diseases involved with abnormal regulation of MMPs have attracted the attentions of physicians, medical scientists and pharmaceutical developers. In Brunch’s membrane, amounts of MMPs such as MMP-2 and MMP-9 were observed to increase and the degree of covalent modification was enhanced for these MMPs with age [6, 7]. The aging changes in the MMP system were shown to be aggressively advanced in age-related macular degeneration, resulting in a large accumulation of abnormal ECM material [8]. Over-expression of MMPs can cause Alzheimer’s disease, a progressive neurologic disorder commonly affecting people over the age of 65 [9]. A recent study showed that the alteration of amyloid precursor proteolysis, a hallmark of Alzheimer's disease, was mediated by MMP-14 in human neuronal cells [10]. Parkinson's disease, which ordinarily begins in late life, has been reported to be associated with aberrant expression of MMP-3 and MMP-9 [11, 12]. MMPs like MMP-2, MMP-9 and MMP-13 have been shown to participate in bone formation and development, and abnormal regulation of these genes contributes to the development of bone diseases [13]. Cancers, occurring mostly in middle age and above, have been linked to MMPs with evidence as they are an important factor promoting tumor progression [14]. This is especially true for tumors like pancreatic adenocarcinoma, prostate adenocarcinoma, bladder urothelial carcinoma and melanoma, which are commonly at risk above the age of 50 [15–18]. Collectively they reveal that MMPs widely participate in physiological and pathological processes, and thus are promising targets for diagnosis and drug development of aging-related diseases.

Twenty-four MMP genes are present in the human genome; among them, MMP-23A is a duplicated copy of MMP-23B and likely a pseudogene [19]. Based on biochemical properties, MMPs can be classified into collagenases (including MMP-1, MMP-8 and MMP-13, whose key feature is the ability to cleave interstitial collagens), stromelysins (MMP-3, MMP-10, and MMP-11), gelatinases (MMP-2 and MMP-9, which can digest gelatins), matrilysins (MMP-7 and MMP-26, which can digest ECM components and activate proMMPs), membrane-type MMPs (MMP-14, MMP-15, MMP-16, MMP-17, MMP-24, and MMP-25, which can digest ECM molecules) and unclassified MMPs (MMP-12, MMP-19, MMP-20, MMP-21, MMP-23, MMP-27, and MMP-28) (Table 1) [20]. Among the unclassified MMPs, MMP-19 is used as a T-cell–derived autoantigen from patients with rheumatoid arthritis, but its exact function is not fully characterized [21]. MMP-20 can digest amelogenin [22]. The limitation of this classification is obvious, as multiple substrates have been revealed for several MMPs. For example, MMP-1, a collagenase, can also cleave tenascin and aggrecan. MMP-14, biochemically classified as a membrane-type MMP, serves as a collagenase, too [23]. Another weakness is that several (7) MMPs are still not classified. These have limited the accuracy of this classification method. An alternative classification was proposed based on domain organization, which categorizes MMPs into four groups, namely archetypal MMPs, matrilysins, gelatinases and furin-activatable MMPs (Table 1) [24]. This classification approach still holds weaknesses as domains defined in this context are not so common as conserved domains commonly referred to like Pfam domains. Therefore, careful investigation is required when allocating a new MMP gene into this classification [25].

**Table 1:** Classification of matrix metalloproteinase (MMP) enzymes.

**Class**	**Type**	**Name**
Archetypal MMP	Collagenase	MMP-1, MMP-8, MMP-13
	Stromelysin	MMP-3, MMP-10
	Others	MMP-12, MMP-19, MMP-20, MMP-27
Gelatinase		MMP-2, MMP-9
Matrilysin		MMP-7, MMP-26
Furin-activatable MMP	Secreted	MMP-11, MMP-21, MMP-28
	Type I transmembrane MT-MMP	MMP-14, MMP-15, MMP-16, MMP-24
	GPI-anchored MMP	MMP-17, MMP-25
	Type II transmembrane MT-MMP	MMP-23A, MMP-23B

In this article, we investigate primate MMPs with evolutionary analyses together with gene structure, genome organization and comparative gene expression analyses. We compare our results with the two stereotypical classification approaches, in the hope to reach consensus for classification approaches. We also identify MMPs of potential value for future studies.

## RESULTS

### Comprehensive identification of putative MMPs from 11 primate genomes

**Figure 1:**
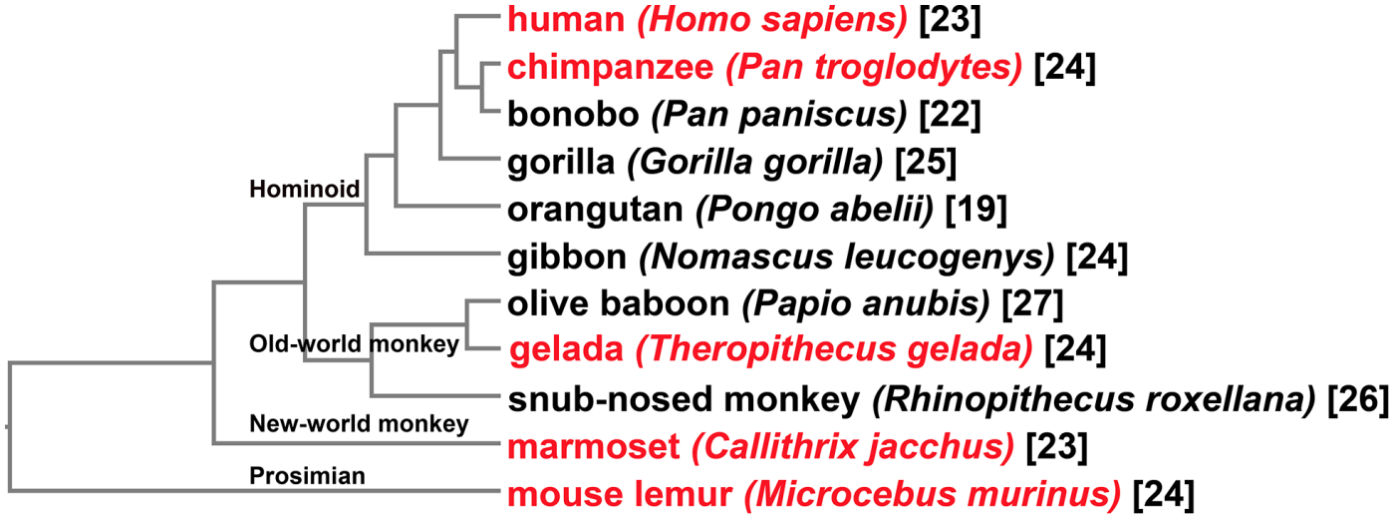
**Taxonomy of the primate species for investigation of MMPs.** At the tip of each clade shows the common name followed by the Latin name of the organism. Tips shown in red represent species with comparative gene expression analyses performed. Shown at nodes are four major primate groups. Numbers in brackets are counting of MMPs identified for each organism.

Primates arose 63–74 million years ago from small terrestrial mammals [26]. To make sure major lineages of primates are covered, 11 species were chosen for identification of putative MMPs in this manuscript (Figure 1). In addition to the human genome, those of bonobo (*Pan paniscus*), chimpanzee (*Pan troglodytes*), gorilla (*Gorilla gorilla*), orangutan (*Pongo abelii*) and gibbon (*Nomascus leucogenys*) were included to represent Hominoids. Olive baboon (*Papio anubis*), gelada (*Theropithecus gelada*) and golden snub-nosed monkey (*Rhinopithecus roxellana*) were sampled to represent old-world monkeys. Marmoset (*Callithrix jacchus*) and mouse lemur (*Microcebus murinus*) represented new-world monkey and prosimian, respectively. All the genome assemblies are chromosome-level, except that of marmoset which has only scaffold-level assembly available.

Through a combination of Basic Local Alignment Search Tool-Protein (BLASTp) and domain-search-based HMMER, a total of 261 putative MMPs were identified (Figure 1). Looking at gene counting in genomes, 19 to 27 MMPs were obtained for each species. Three Hominini species, human, chimpanzee and bonobo, harbor 23, 24, and 22 genes, respectively. Three other Hominoid species, gorilla, orangutan, and gibbon contain 25, 19 and 24 MMPs, respectively. Compared to other primate groups, MMP copy numbers in old-world monkey genomes are generally greater, with 27, 24 and 26 genes in olive baboon, gelada and snub-nosed monkey, respectively. In marmoset, a new-world monkey species, 23 MMPs were obtained. Mouse lemur, one of the smallest living primates, harbor 24 MMPs.

### Classification of primate MMPs based on phylogenetic inference

**Figure 2:**
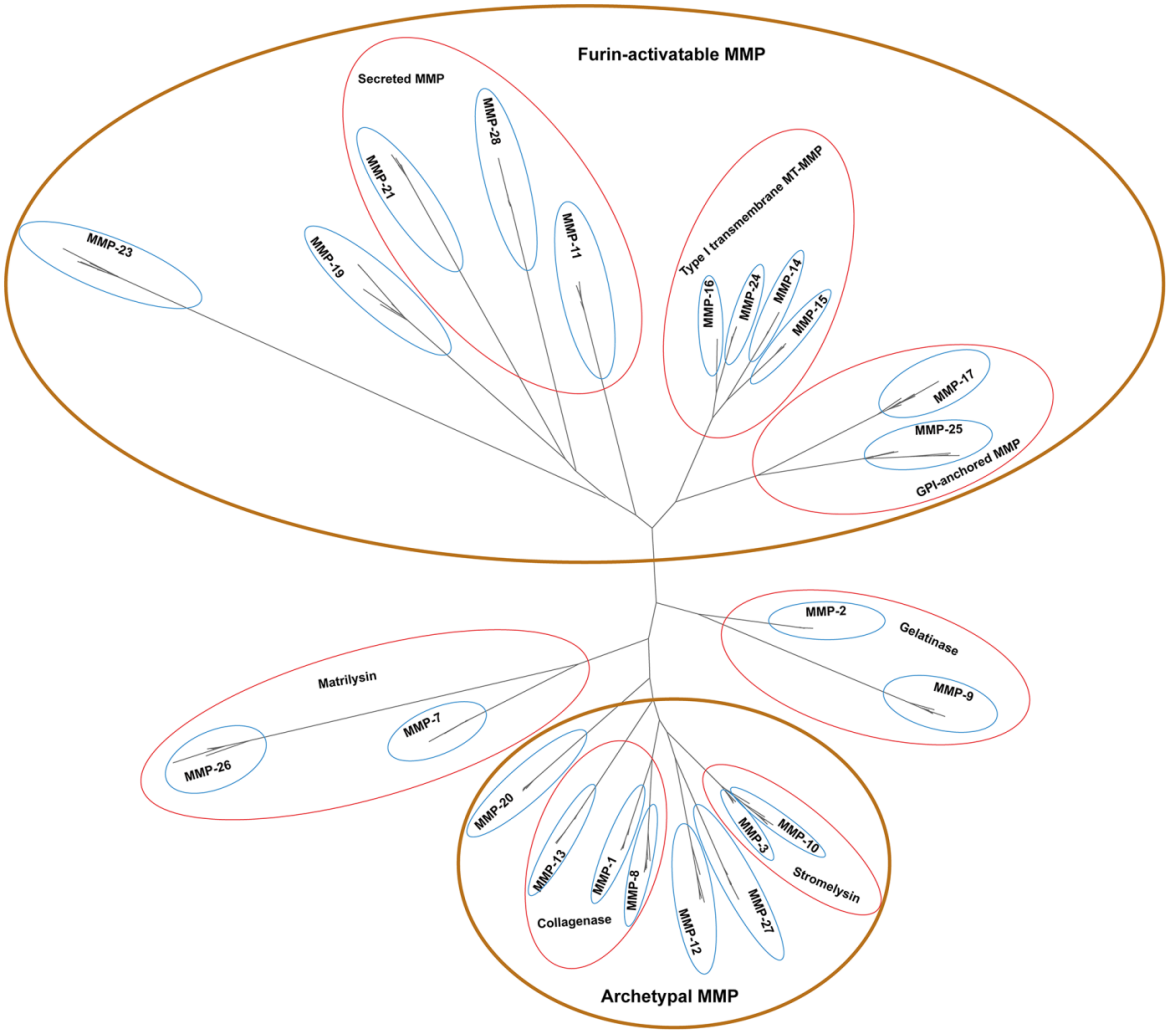
**Unrooted phylogenetic tree of 261 primate MMP peptides.** Looped in brown ovals are four major groups supported with biochemistry evidence, and enclosed in red circles are sub-groups based on biochemical properties under certain groups. Phylogenetic analyses were performed by using PhyML v3.3.3 with 1000 bootstraps.

By developing a phylogenetic tree, the peptides were classified into 23 distinct clades, with high bootstrap support at basal nodes (Figure 2). One human MMP was categorized into each clade, confirming the reliability of the tree topology. Each of the 23 MMP genes was identified in each of the most genomes, suggesting conserved evolution of the MMP family in primates (Supplementary Table 1). In this manuscript, very limited number of genes were missing in certain clades. For example, MMP-3 was failed to identify in mouse lemur, and MMP-7 was not retrieved from the gorilla genome. This result indicates a limited number of MMPs are missing from certain primate genomes, but the possibility of genome assembly errors or annotation artifacts could not be ruled out.

**Figure 3:**
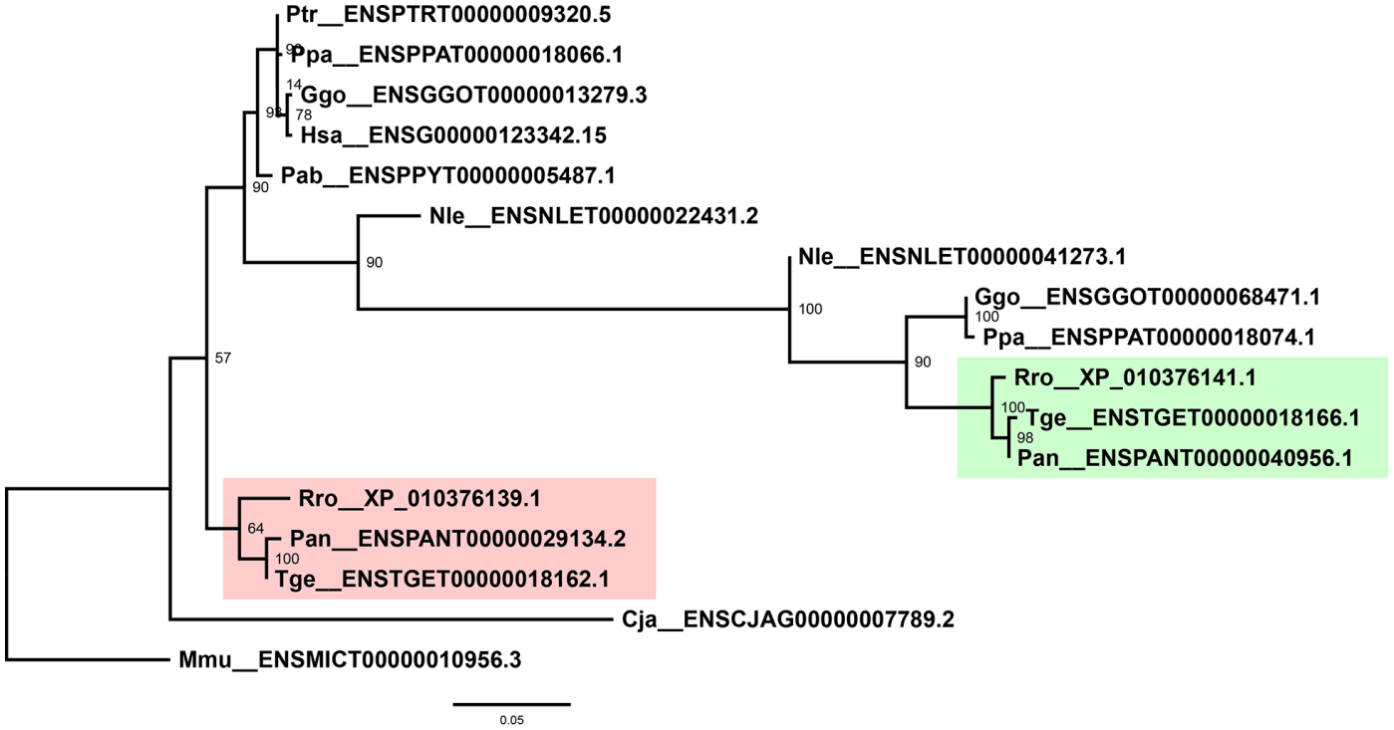
**Phylogenetic tree of primate MMP-19s.** Shaded are two independent clades as a result of duplication of old-world monkey MMP-19s. Labeled at nodes are bootstrap support values. Phylogenetic analyses were performed by using PhyML v3.3.3 with 1000 bootstraps.

Interestingly, two copies of MMP-19 were present in each of the old-world monkey genome. To resolve relationships of these genes, phylogenetic trees of the MMP-19 peptides alone were reconstructed, which resolved the two copies into two distinct clades with high bootstrap support (Figure 3). In each clade, 1 copy of MMP-19 was represented for each of the three species, suggesting duplication of MMP-19 at the early stage of or before the emergence of old-world monkeys.

Based on biochemical properties, MMPs can be classified into four groups, namely archetypal MMPs, matrilysins, gelatinases, and furin-activatable MMPs (Table 1) [24]. Our phylogenetic topology shows that MMPs are clustered in four major groups (shown in brown circles in Figure 2). MMP-1s, MMP-8s and MMP-13s are proximate to each other, which biochemically serve as collagenases; MMP-3 and MMP-10, two clades as stromelysins, form an independent group. These peptides, together with MMP-12s and MMP-27s, are clustered together, which can be biochemically classified as archetypal MMPs. MMP-11s, MMP-21s and MMP-28s, three secreted MMPs, are proximate to each other. MMP-14s, MMP-15s, MMP-16s and MMP-24s, which are type-I transmembrane MT-MMPs, form a group. MMP-17 and MMP-25, GPI-anchored MMPs, clustered together. These peptides, together with MMP-19 and MMP-23, form a group and can be classified as furin-activatable MMPs, except MMP-19 which is referred to as an archetypal MMP-. MMP-7s and MMP-26s serve as matrilysins, and MMP-2s and MMP-9s serve as gelatinases, and they form a group, respectively. In summary, the phylogenetic topology of primate MMPs largely agrees with biochemistry-based classification, which demonstrates the power of phylogenetic analysis as a guide in biochemical characterization of a certain MMP gene.

### Evolution of primate MMPs

To investigate the possibility of positive selection among primate MMPs, a maximum-likelihood-based estimation of codon substitution model was carried out, on the basis of codon-enabled sequence alignment. Hypothesis of positive selection was tested against purifying selection with a likelihood ratio test (LRT). Overall, primate genes of MMP-8, MMP-9, MMP-10, MMP-19 and MMP-26 showed positive selection, with the M8 model fitting the data significantly better than the M7 model (Table 2). Furthermore, predicted ω for each site was calculated as the weighted mean of ω categories, and candidate sites of positive selection were detected in MMP-8, MMP-10, MMP-19 and MMP-26. The excess of candidate sites of positive selection is striking for MMP-19, with 12 positive selected sites detected (Table 2).

**Table 2:** Test of positive selection among clades of primate MMPs.

	**lnL(M7)**	**lnL(M8)**	***P*-value**	**Positive selection sites**
MMP-1	-3332.31	-3331.97	0.713	-
MMP-2	-3230.67	-3229.95	0.486	-
MMP-3	-3662.88	-3662.19	0.499	-
MMP-7	-2007.11	-2004.66	0.086	-
MMP-8	-3203.69	-3195.15	0.000	H35,G67,T124
MMP-9	-5414.77	-5408.82	0.003	-
MMP-10	-3099.71	-3095.91	0.022	G113
MMP-11	-2908.63	-2907.80	0.435	-
MMP-12	-3597.70	-3597.22	0.619	-
MMP-13	-3276.51	-3276.22	0.747	-
MMP-14	-3505.05	-3505.05	1.000	-
MMP-15	-4486.82	-4486.59	0.794	-
MMP-16	-3171.86	-3171.86	0.999	-
MMP-17	-3222.31	-3220.89	0.242	-
MMP-19	-1882.99	-1874.37	0.000	P93,A98,G122,L135,P164,L186, G187,L189,F190,S196,Y203,T206
MMP-20	-3309.21	-3307.24	0.140	-
MMP-21	-4067.37	-4067.37	1.000	-
MMP-23	-1816.71	-1816.71	1.000	-
MMP-24	-2961.92	-2960.96	0.385	-
MMP-25	-1513.27	-1513.14	0.874	-
MMP-26	-1531.90	-1527.78	0.016	M6
MMP-27	-3414.02	-3412.24	0.168	-
MMP-28	-3443.36	-3443.19	0.844	-

To further investigate molecular evolution of human MMPs, evolutionary rates between human and chimpanzee orthologs were calculated. Results showed that *Ka* (nonsynonymous substitution), *Ks* (synonymous substitution) and *Ka/Ks* showed variation among orthologs of MMPs. Only MMP-26 showed *Ka/Ks* ratio above 1, implying selection of human MMP-26 gene (Table 3). MMP-17 showed the highest synonymous substitution rate (*Ks* = 0.0398), and MMP-3, MMP-11 and MMP-15 also showed *Ks* values above 0.025, suggesting relatively relaxed pressure which results in sequence divergence for these genes. Notably, orthologs of MMP-24 showed 0 value of *Ka*, which indicates that function of human MMP-24 is strictly restricted.

**Table 3:** Nonsynonymous and synonymous substitutions between human and chimpanzee MMP orthologs.

**MMP**	**human**	**chimpanzee**	***Ka***	***Ks***	***Ka/Ks***
MMP-1	ENSG00000196611.4	ENSPTRT00000007870.6	0.0031	0.0071	0.444
MMP-2	ENSG00000087245.12	ENSPTRT00000092414.1	0.0007	0.0247	0.027
MMP-3	ENSG00000149968.11	ENSPTRT00000007868.3	0.0063	0.0295	0.2139
MMP-7	ENSG00000137673.8	ENSPTRT00000007862.4	0.0075	0.0242	0.3092
MMP-8	ENSG00000118113.11	ENSPTRT00000007866.5	0.0071	0.0156	0.4591
MMP-9	ENSG00000100985.7	ENSPTRT00000025282.4	0.0024	0.0117	0.2077
MMP-10	ENSG00000166670.9	ENSPTRT00000007867.5	0.0049	0.0127	0.3881
MMP-11	ENSG00000099953.9	ENSPTRT00000026441.6	0.0029	0.0261	0.1099
MMP-13	ENSG00000137745.11	ENSPTRT00000088050.1	0.0065	0.0217	0.3017
MMP-14	ENSG00000157227.12	ENSPTRT00000011266.6	0.0008	0.0225	0.0356
MMP-15	ENSG00000102996.4	ENSPTRT00000015071.5	0.0019	0.0279	0.0679
MMP-16	ENSG00000156103.15	ENSPTRT00000099969.1	0.0015	0.0174	0.086
MMP-17	ENSG00000198598.6	ENSPTRT00000010368.6	0.0035	0.0398	0.0889
MMP-19	ENSG00000123342.15	ENSPTRT00000009320.5	0.0029	0.0109	0.2691
MMP-20	ENSG00000137674.3	ENSPTRT00000007863.3	0.0041	0.0089	0.4612
MMP-21	ENSG00000154485.4	ENSPTRT00000005789.2	0.0025	0.0122	0.2076
MMP-23	ENSG00000189409.13	ENSPTRT00000000071.5	0.001	0.0239	0.0404
MMP-24	ENSG00000125966.9	ENSPTRT00000024960.5	0	0.0119	0.001
MMP-25	ENSG00000008516.16	ENSPTRT00000014165.7	0.0038	0.0089	0.4229
MMP-26	ENSG00000167346.7	ENSPTRT00000006170.3	0.0126	0.0036	3.5059
MMP-27	ENSG00000137675.4	ENSPTRT00000007864.3	0.0061	0.0135	0.4547
MMP-28	ENSG00000271447.5	ENSPTRT00000045218.3	0.0026	0.014	0.1825

### Conservation and divergence of domain organization and gene structure in primate MMPs

**Figure 4:**
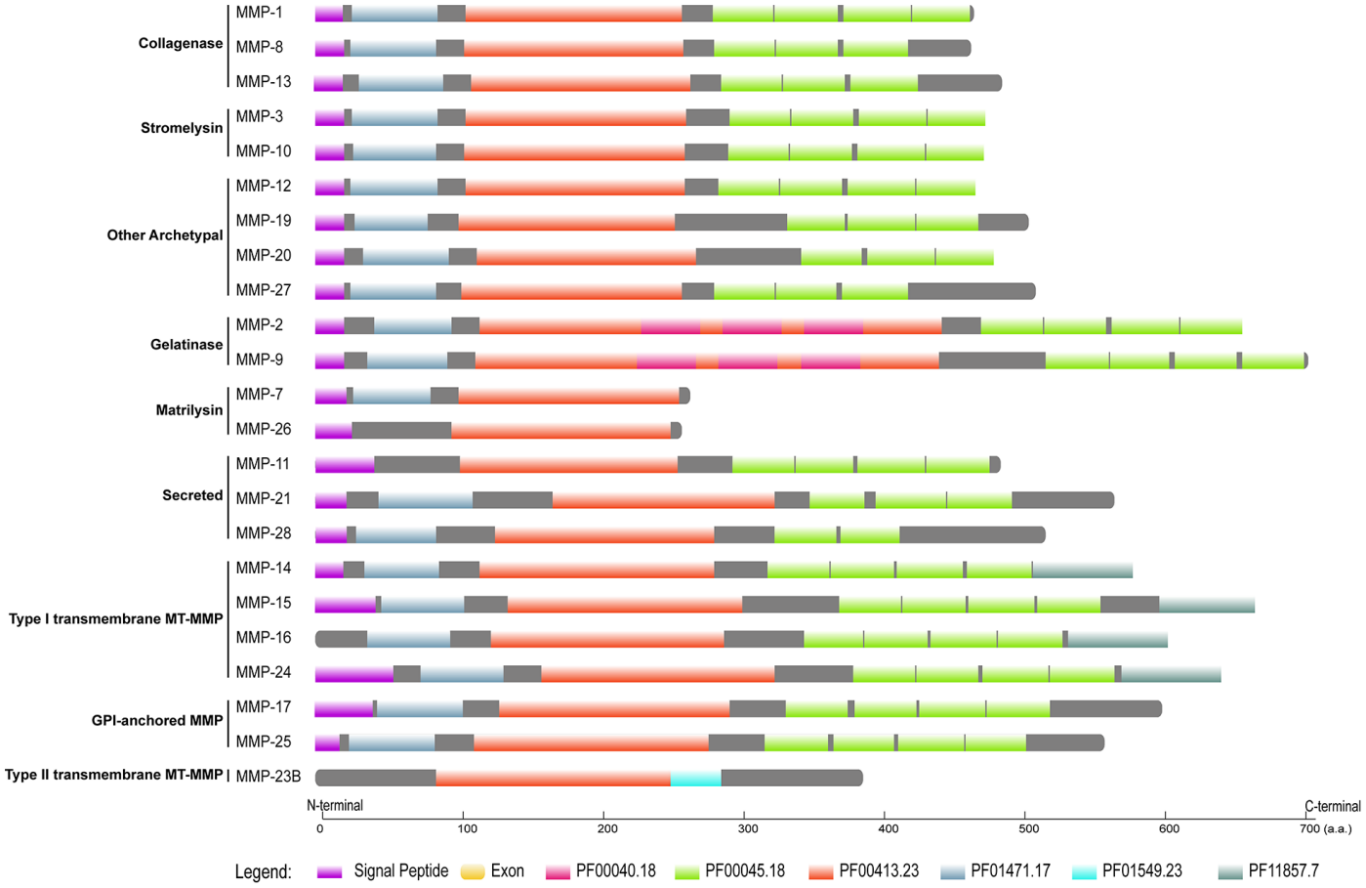
**Domain organization of human MMPs.** Pfam domains were identified by using HMMER 3.3 against Pfam database, and signal peptides were obtained by querying SignalP-5.0 server. Pfam domains and signal peptides are shown as colored boxes.

Like those of many other families, MMP peptides contain a conserved domain structure, which has been revealed in human and many other organisms [19, 24, 27]. By querying the databases of Pfam and SignalP, we identified a cascade of domains in each MMP peptide (Figure 4; function information is described in Supplementary Table 2). All the MMPs contain a domain with Pfam ID PF00413, which is a catalytic metalloproteinase domain (Supplementary Table 2). Near the N-terminal, there is a domain resembling bacterium peptidoglycan binding domain (PF01471) for all the MMPs except MMP-11, MMP-26 and MMP-23B. At the C-terminal, hemopexin-like repeats were contained in all MMPs except MMP-23B and the two matrilysins. A signal peptide was identified in all MMPs except MMP-16 and MMP-23B. In many non-human primate species, each gene shares the same domain organization like that in human, except limited peptides (Supplementary Table 3). For example, for MMP-24, only the human and chimpanzee sequences have the signal peptide, and we failed to identify it in all the other primate species. Most conserved domains we failed to identify were located at N- or C-terminal, suggesting that they are results of genome annotation artefacts.

**Figure 5:**
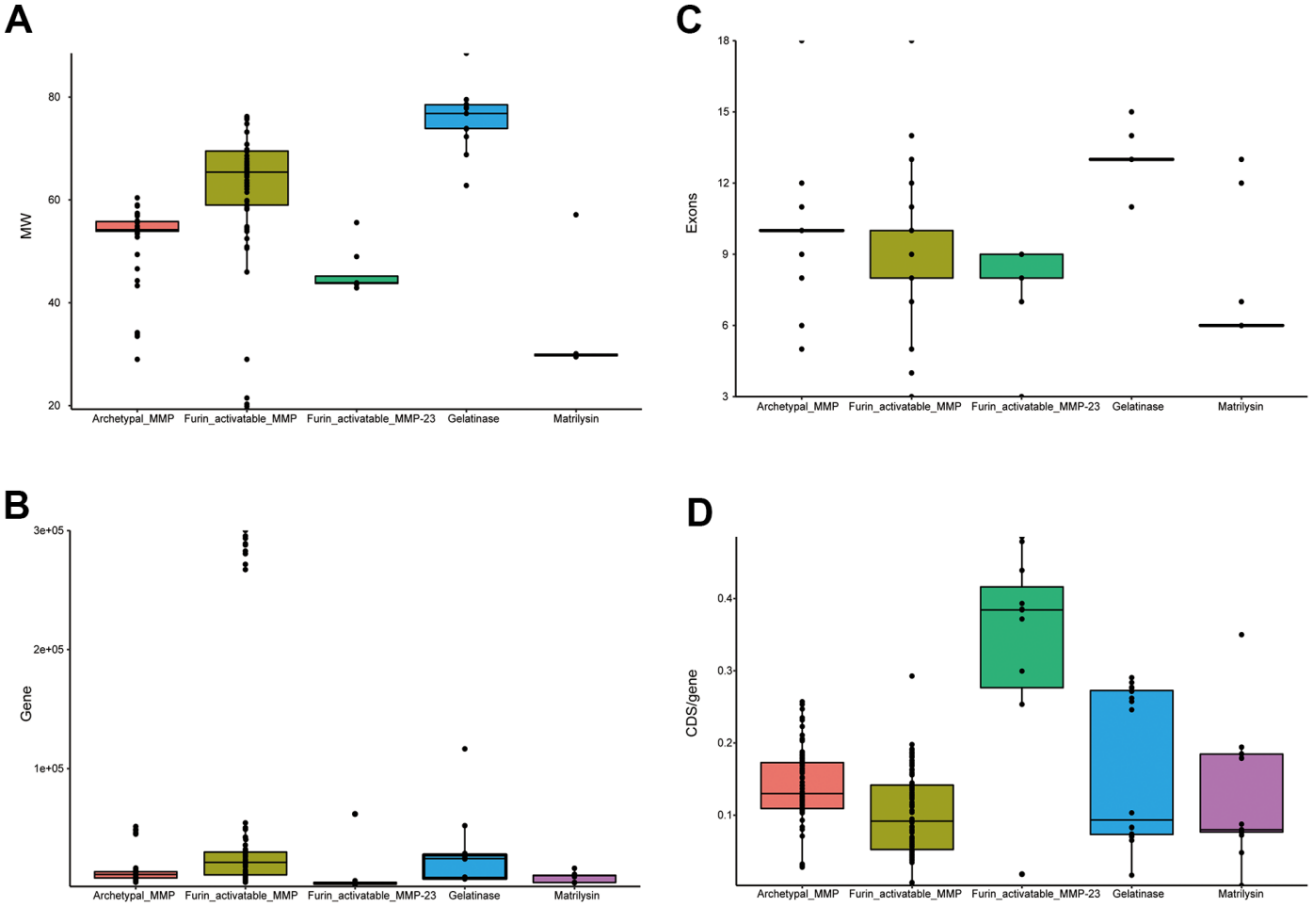
**Statistics of primate MMP gene structure.** (**A**) Box plots of molecular weights of MMP peptides. (**B**) Box plots of gene lengths. (**C**) Box plots of CDS/gene ratio. (**D**) Box plots of exon counting. While MMP-23s are classified in furin-activatable MMPs, they demonstrate very different characteristics than other MMPs, and thus were investigated separately.

As the domain organization is different, we investigated gene structure among MMPs. Gelatinases (MMP-2 and MMP-9) have the largest molecular weights, which might be attributable to the extra domain (PF00040) they have. Matrilysin (MMP-7 and MMP-26) show the smallest molecular weights, as they have the simplest domain organization; they have the largest CDS (coding sequence)/gene ratios, which demonstrates that they have the most compact gene structure. Furin-activatable MMPs are the longest genes, and CDS/gene ratios are low, which indicates that they have the longest introns. Exon numbers vary both among the different MMP types and within the same type; exon numbers of gelatinases are overall the largest. Among the groups of MMPs, furin-activatable MMPs show the largest variation for molecular weight, gene length and exon counting (Figure 5).

### Genomic organization of primate MMPs

**Figure 6:**
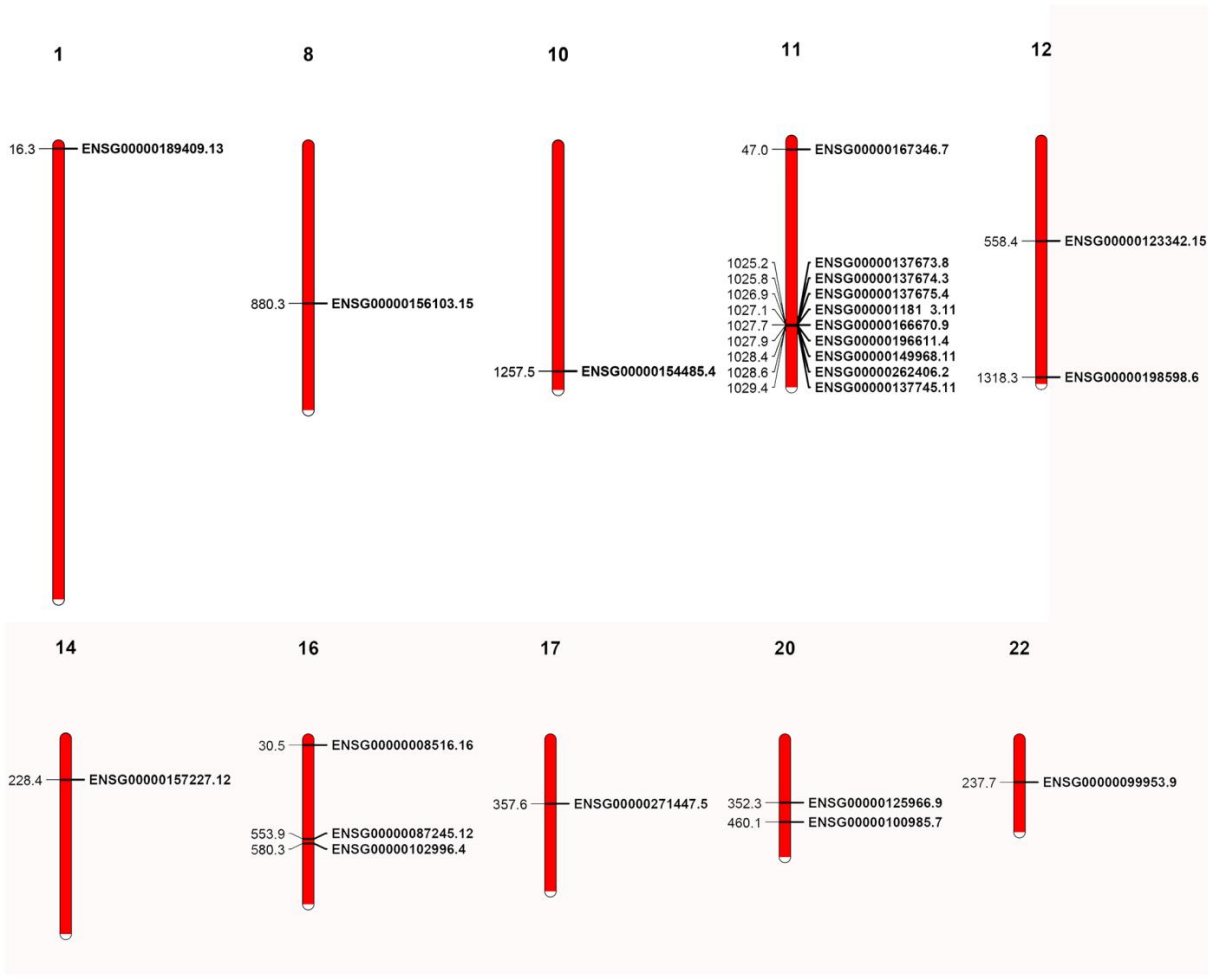
**Localization of human MMP genes.** A cluster is shown in the 11th chromosome. The localization of the genes was retrieved from GFF3 files.

As gene sequence, domain organization and gene structure of the MMP genes show both conservation and divergence, we investigated the conservation of genomic organization for the primate genes. We first mapped the human MMP genes to chromosomes and discovered that the 23 human genes are located at 10 chromosomes. Interestingly, 9 MMP genes are located together as a single cluster in the 11th chromosome, of which most are archetypal MMPs (MMP-1, -3, -8, -10, -12, -13, -20, and -27), and one encodes matrilysin (MMP-7) (Figure 6).

**Figure 7:**
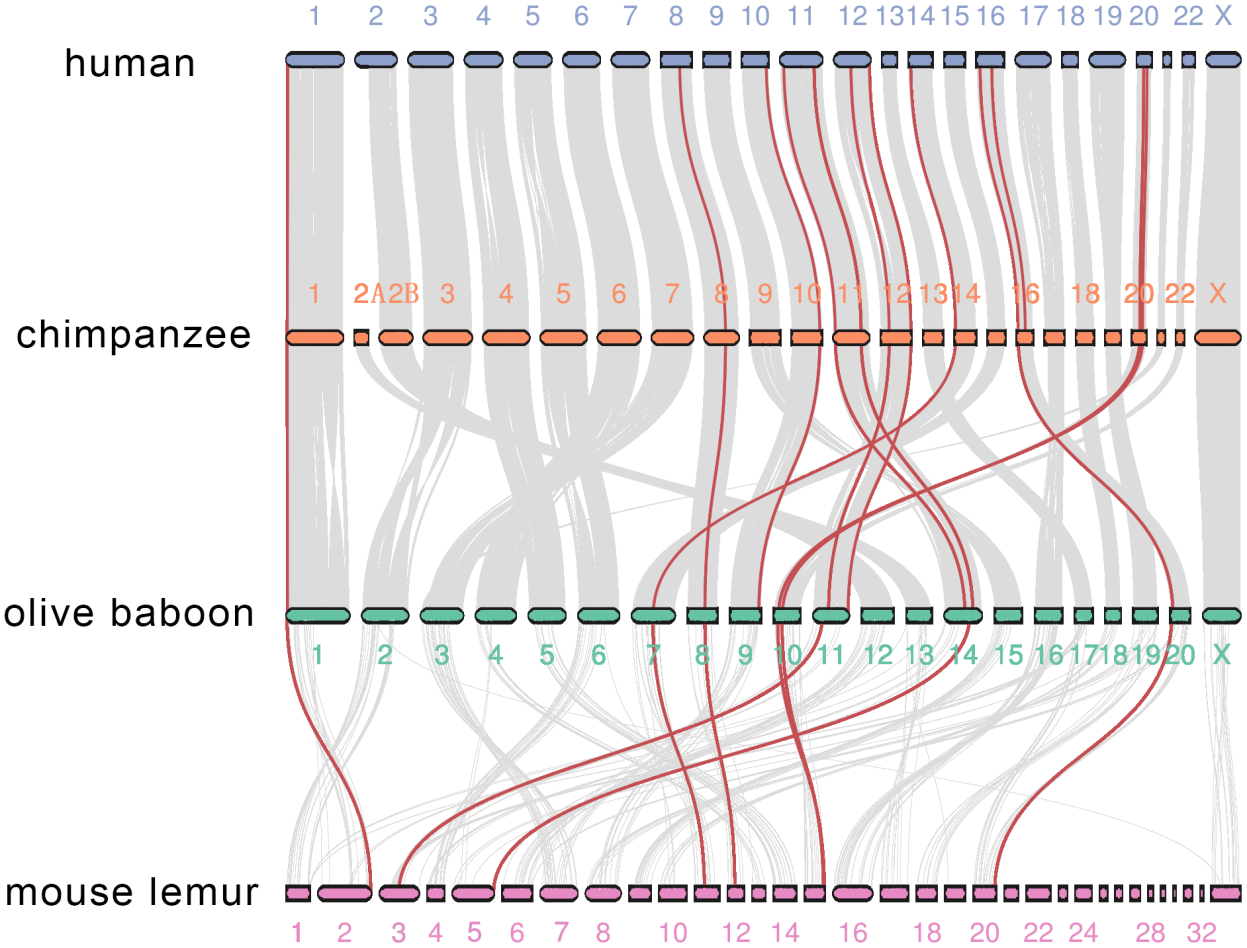
**Co-synteny of the MMP genes across human, chimpanzee, olive baboon and mouse lemur.** Co-synteny clusters are shown as grey curves, and MMPs in the co-synteny clusters are shown in red.

We then investigated co-synteny of the primate genomes and the MMPs in the co-synteny clusters. We compared the human genome with the genomes of three other primates, chimpanzee, a Hominoid, olive baboon, an old-world monkey, and mouse lemur, a Prosimian species. The genome of marmoset, the only new-world monkey representative, was not assembled to the chromosome level, and thus was excluded from the co-synteny analysis [28]. There was high co-synteny between the human and chimpanzee genomes, and between chimpanzee and olive baboon genomes. There was moderate co-synteny between the olive baboon and mouse lemur genomes. Twenty-one of 23 human genes are located in 12 co-synteny clusters. A cluster in the human 16th chromosome, where MMP-2 and -15 are located, is not shared between chimpanzee and olive baboon. Only 16 MMPs are within the 8 co-synteny clusters between olive baboon and mouse lemur. The co-synteny cluster in the human 11th chromosome, where the 9-gene cluster is located, is conserved across all the four primate species (Figure 7). Together, these results demonstrate that both conservation and divergence of MMPs are attributable to the conserved genome organization over primates and changes of co-synteny as the genetic relationship gets further.

### Expression of MMP genes in human and across primates

**Figure 8:**
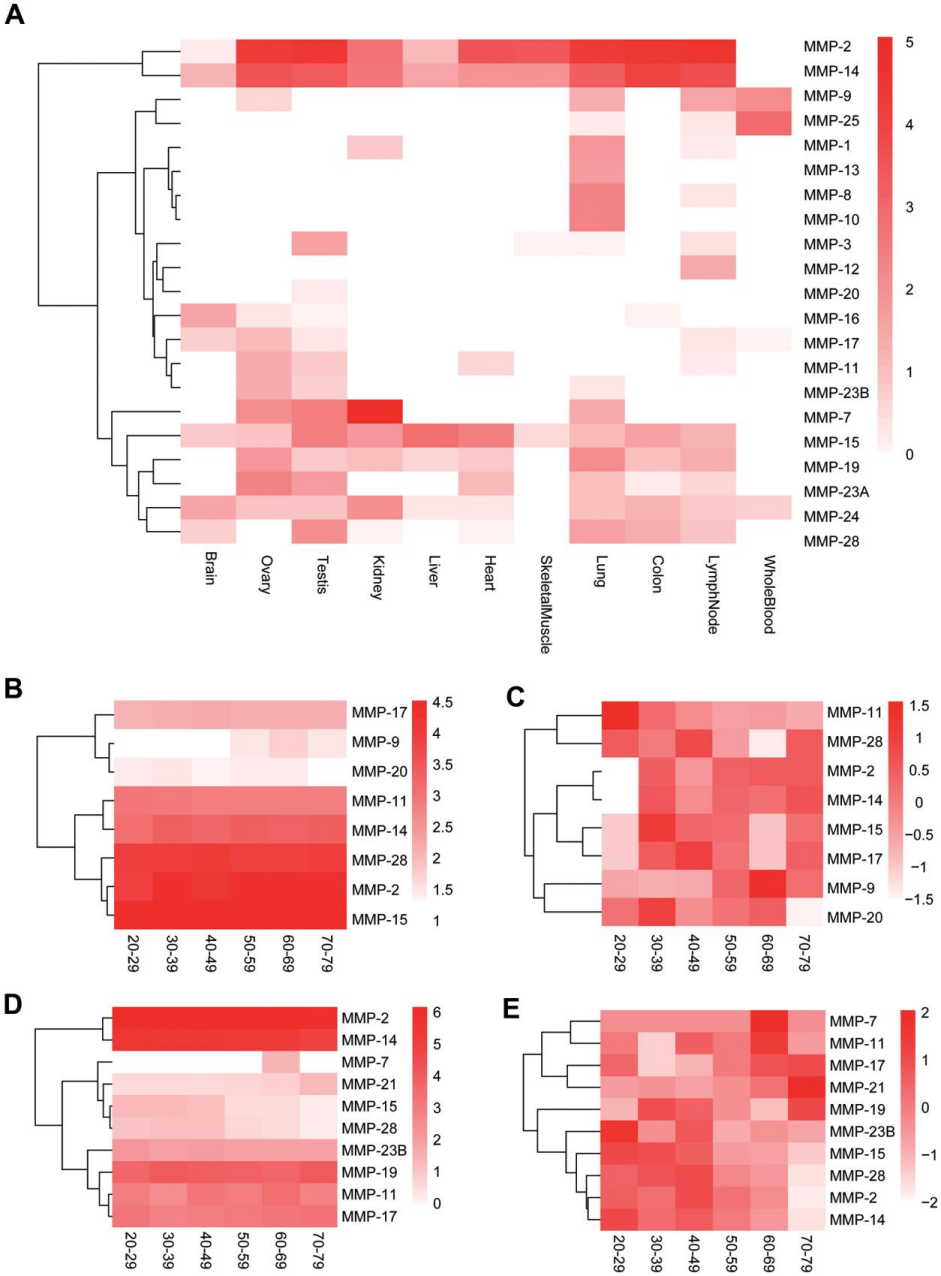
**Expression of human MMP genes.** (**A**) Heatmap of human MMP gene expression in 11 tissues. (**B**) Longitudinal expression of human MMPs in testis (not normalized). (**C**) Longitudinal expression of human MMPs in testis (normalized). (**D**) Longitudinal expression of human MMPs in ovary (not normalized). (**E**) Longitudinal expression of human MMPs in ovary (normalized). The MMP genes are clustered based on expression patterns.

As MMPs are clustered into different groups, little is known about the relatedness among the MMPs on the gene expression level. We extracted MMP gene expression data of 11 human tissues from Human BodyMap and performed co-expression analysis among the genes. Gene expression data show that, although genes like MMP-2 and MMP-14 show ubiquitous high expression, many genes have tissue-specific expression patterns, and MMPs are expressed in all tissues except skeletal muscles, in which only limited genes like MMP-2 and MMP-14 are expressed (Figure 8A).

MMPs play important roles in human aging [29]. As effects of aging are particularly evident on both male and female reproductive system [30, 31], we further investigated longitudinal expression of the MMP genes in male and female reproductive organs, testis and ovary, respectively, by using GTEx data [32]. In testis, the male sexual organ, MMP-2, MMP-11, MMP-14, MMP-15, MMP-17 and MMP-28 are highly or moderately expressed across all ages from 20-79 (Figure 8B). MMP-11 showed gradually decreased expression during human aging, and all the other moderately or highly expressed genes showed lower expression at the ages of 20-29 than the older ages in testis (Figure 8C). In ovary, the female sexual organ, MMP-2, MMP-11, MMP-14, MMP-17 and MMP-28 showed universally moderate or high expression across all ages, the same as those in testis; in addition, MMP-19 and MMP-23B were highly expressed, too, which is different to those in testis (Figure 8C). Two lowly-expressed genes, MMP-7 and MMP-21, showed elevated expression during aging, and MMP-2, MMP-14, MMP-15 and MMP-28 showed the reversed trend in ovary (Figure 8D).

**Figure 9:**
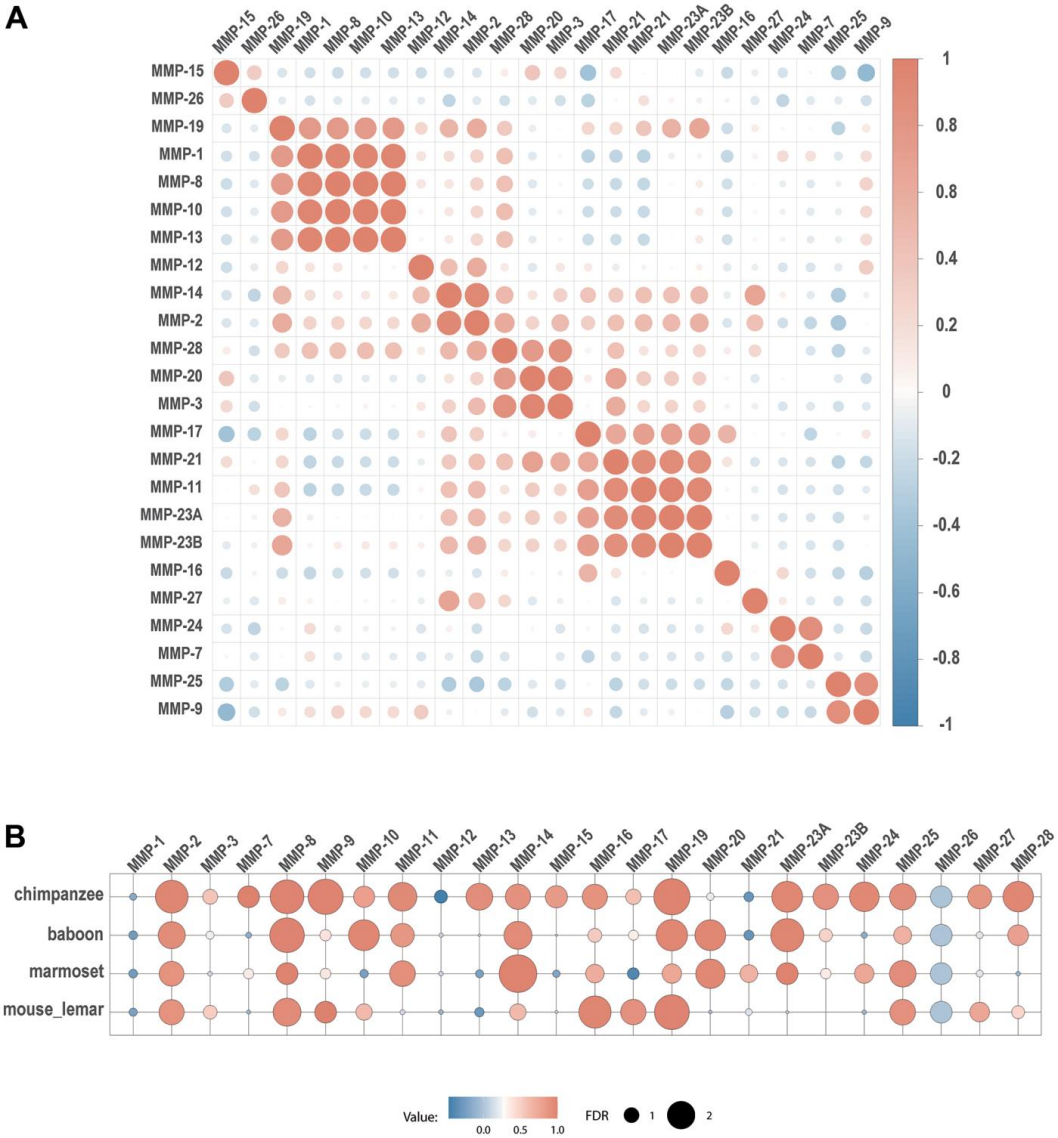
**Co-expression of MMPs.** (**A**) Expression correlation between human MMP genes. (**B**) Expression correlation of each MMP gene between human and four other primate species. Spearman’s correlation coefficients are depicted as heatmap, with each circle denoting a pair.

Co-expression analysis shows that all the three genes encoding collagenases (MMP-1, MMP-8 and MMP-13) are co-expressed (Figure 9A). In addition, they formed a co-expression cluster with MMP-10 and MMP-19. Interestingly, the expression of some genes is correlated even they belong to different groups based on stereotypical classifications. For example, a gene cluster formed for five furin-activatable MMP genes, MMP-11, MMP-17, MMP-21, MMP-23A and MMP-23B, although they belong to different groups according to biochemistry-based classification. In addition, groups were formed for genes in different groups by domain organization, such as pair of MMP-7 and MMP-24, and pair of MMP-9 and MMP-25 (Figure 9A).

To explore the relationship of gene expression patterns across primates, we compared gene expression of human MMP genes with those in chimpanzee, olive baboon, marmoset and mouse lemur. Between human and chimpanzee, 13 MMP genes (MMP-2, MMP-7, MMP-8, MMP-9, MMP-11, MMP-13, MMP-14, MMP-16, MMP-19, MMP-23A, MMP-23B, MMP-24, MMP-28) demonstrated co-expression. Fewer co-expression pairs were obtained between human and other species having longer genetic distance. Only 7 genes (MMP-2, MMP-8, MMP-9, MMP-16, MMP-17, MMP-19 and MMP-25) showed as co-expression pairs.

Expression of MMP-2 and MMP-8 showed the most conserved, as they showed co-expression between human and all the four non-human primate species (Figure 9B).

## DISCUSSION

Non-human primates are essential models for modern medical and biochemical studies, because of their genetic, physiological and psychological similarity to humans. Even mouse lemur, which is placed at the basal node of primate taxonomy and roughly half the genetic distance between mice and humans, has been shown as a suitable model organism for drug development and genetics studies [33]. Investigation of gene evolution among primates can shed light on biochemical role, physiological and pathological functions of a certain gene, gene family, signaling network or metabolic pathway, and can offer insights in the discovery of prognostic biomarkers and therapeutic targets, which have been demonstrated in numerous articles (for example, [34–39]). In this article, we provide an example of how this can be utilized in the analysis of the MMP family enabled by the high-quality genomes across primates.

MMPs comprise a family of zinc-dependent endoproteinase, responsible for degrading extracellular matrix components [1]. Deregulation of the MMPs has been implicated in many aging-related diseases, such as Alzheimer’s disease, Parkinson's disease, age-related macular degeneration, bone diseases and cancers [6, 9, 11–13, 15–18]. Although biochemical properties of MMPs has been intensively studied, accumulated evidences have shown that our understanding of the MMPs is far from absolute, and some discoveries even have conflicted conclusions with previous findings [23, 40]. In this article, we comprehensively investigate the MMP family across the primates from the perspectives of phylogeny, sequence divergence, domain organization, gene structure, genome organization and gene expression. To our knowledge, this is the first comprehensive investigation of MMPs across primates.

We identified signal peptides at the N-termini and Pfam domains at the C-termini in most MMP peptides. Very limited peptides have conserved domains failing to identify, most of which are located at N- or C-termini (Supplementary Table 2). One possible reason is that they represent bona fide loss of the conserved domains during the evolution of certain primate species. But there is a possibility of incomplete genome assembly and genome annotation artifact which can result in incomplete sequence prediction of these MMPs. This is very common in eukaryotic genome assembly and annotation despite rapid advancement of genome sequencing technology [41, 42].

Our results showed that furin-activatable MMPs are the most variable among different classes of MMPs, including sequence divergence inferred by phylogeny, domain organization and gene structure. There are four types of MMPs within this class, and biochemical properties vary considerably among them [1, 19, 43]. Topology of MMP phylogeny shows that sequence divergence among furin-activatable MMPs is considerably large compared to others. Among the furin-activatable MMPs, MMP-19 showed positive selection in primates with the excess of candidate sites of positive selection. Biochemical and physiological roles of MMP-19 have not been characterized, although MMP-19 is used as a T-cell–derived autoantigen from patients with rheumatoid arthritis [21]. Our results indicate that MMP-19 may have important roles, highlighting its value for further studies. Furthermore, MMP-11, MMP-15 and MMP-17 showed relatively high Ks values than the others.

Our results showed that MMP gene sequences are conserved across primate organisms, and gene organization and genome localization are less conserved as the taxonomic relationships are getting more genetically distant. Additionally, we revealed that MMPs of different types can form a single co-expression cluster. For example, MMP-11 and MMP-21 are secreted MMPs, MMP-17 is a GPI-anchored MMP-, and MMP-23 is a type-II transmembrane MMP- [19] (Figure 9A). Expression patterns of these genes are correlated to each other, suggesting their functional relationships. Another notable co-expression cluster is formed with MMP-1, MMP-8, MMP-10, MMP-13 and MMP-19, all of which are archetypal MMPs. Particularly, all the collagenases (MMP-1, MMP-8 and MMP-13) are co-expressed, which have been shown to play roles of collagenases in wounds and ulcers [44, 45]. These results suggest functional conservation within archetypal MMPs.

Both gelatinases (MMP-2 and MMP-9) are secreted, zinc-dependent endopeptidases and are cell-surface transducers [46]. They are associated with several cancers and thus can be served as cancer biomarkers [46]. Our results showed that the genes are comprised by the longest peptide sequences. However, they showed different characteristics in expression. MMP-2 had high expression across all organs we investigated, while MMP-9 showed tissue-specific expression (Figure 8A). MMP-2 showed conserved expression across all five primate species (Figure 9B), while MMP-9 only shoed expression between human and chimpanzee. These results indicate functional divergence between these two gelatinase genes, which deserve further investigations. In addition to MMP-2, several MMP genes show conserved expression across all five primate species (human, chimpanzee, baboon, marmoset and mouse lemur), including MMP-8, MMP-19 and MMP-25. In addition, MMP-11, MMP-14, MMP-23 showed conserved expression in at least three species when compared to human. Less divergence in expression of these genes imply their conserved function across primates. MMP-24, MMP-25 and MMP-27 showed expression in specific organs; MMP-1, MMP-3, MMP-12, MMP-26 showed very weak conservation among the primates; MMP-3 and MMP-26 also showed divergence in sequence. These results indicate their functional divergence among primates.

Life-history diversity are evident among primates. Human life histories have been described as “slow”, as humans have delayed maturity and lower adult mortality compared to chimpanzee [47, 48]. As MMPs can mediate aging-associated remodeling of extracellular matrix (ECM) [29], we can expect divergence of evolutionary rates among MMPs. In this article, we report positive selection for MMP-26, and evident sequence divergence for MMP-3, MMP-11, MMP-15 and MMP-17. MMP-26 has been indicated to associate with a wide range of pathological process, such as breast cancer, glioma, wounds and ischemic stroke, but lack of evidence highlights necessity of further investigation of this gene [49–52]. We also showed decline of expression for MMP-11 in testis and the same trend for MMP-15 in ovary during human aging. However, expression of MMP-15 and MMP-17 was elevated during aging in testis, suggesting their positive regulatory roles in aging-associated ECM remodeling. These results indicate that certain MMPs such as MMP-11, MMP-15 and MMP-17 may play important roles in life-history variation among primates. Interestingly, several genes including MMP-2, MMP-14 and MMP-15 showed reversed trend expression across ages in testis and ovary; MMP-20 showed longitudinal trend of expression in testis, and MMP-28 only showed trend in ovary. These results suggest sexual difference of functions among these genes.

In addition to cancers, evidence has been reported on associations with psychiatric disorders for some MMPs. For example, MMP-14 can be mediate alteration of amyloid precursor proteolysis, a hallmark of Alzheimer's disease, in human neuronal cells [10]. Parkinson's disease has been associated with aberrant expression of MMP-3 and MMP-9 [11, 12]. Our results showed that MMP genes such as MMP-15, MMP-16, MMP-17, MMP-24 and MMP-28 were expressed highly or moderately in brain, implying the values in investigation of more genes in association with psychiatric diseases.

**Figure 10:**
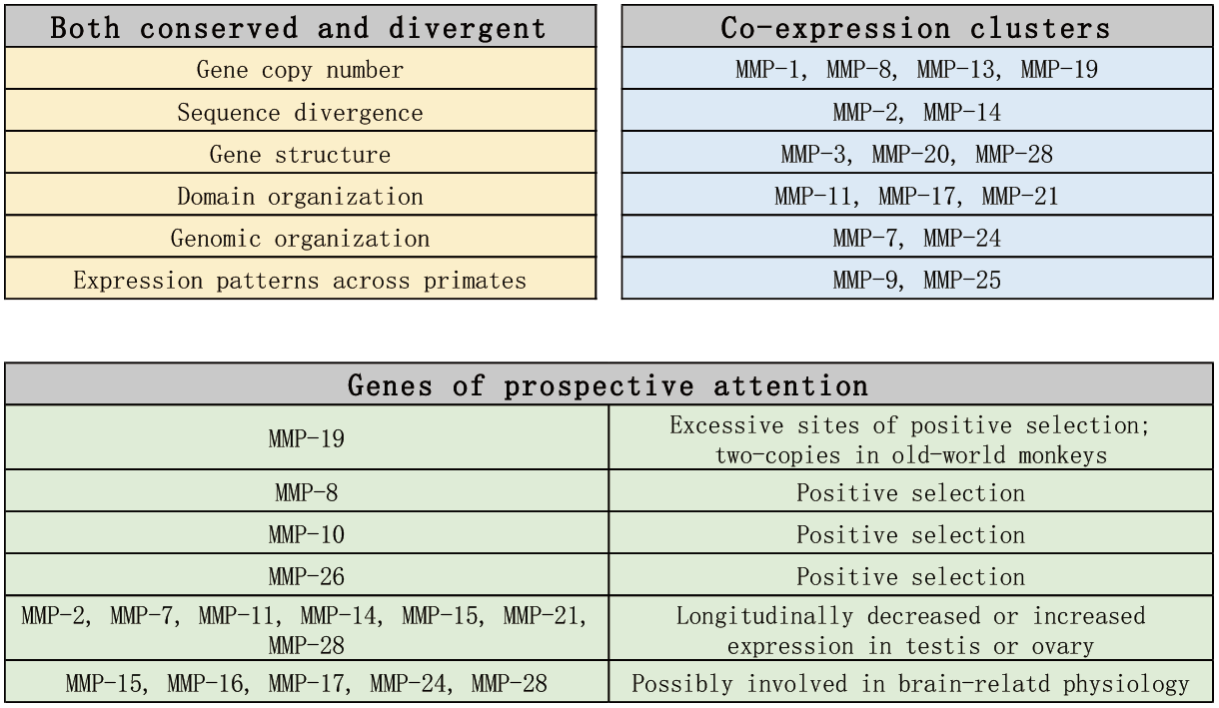
Summary and prospect of the findings in this article.

In conclusion, we carried out genome-enabled identification and characterization of putative MMPs across 11 primate organisms from the perspectives of phylogeny, domain organization, gene structure, genome organization and gene expression, and we identified MMPs which need attentions in future studies (As summarized in Figure 10). Our results shed new light on the classification of MMP family, and our findings can illuminate the discovery of prognostic biomarkers and therapeutic targets for aging-related diseases. [29, 47, 48].

## MATERIALS AND METHODS

### Sources of genome sequence data

Genome sequences, coding sequences, peptides and GFF files were obtained from Ensembl for human (*Homo sapiens*), chimpanzee (*Pan troglodytes*), gelada (*Theropithecus gelada*), orangutan (*Pongo abelii*), olive baboon (*Papio anubis*), bonobo (*Pan paniscus*), gibbon (*Nomascus leucogenys*), mouse lemur (*Microcebus murinus*), gorilla (*Gorilla gorilla*) and marmoset (*Callithrix jacchus*) [53]. Genome files of golden snub-nosed monkey (*Rhinopithecus roxellana*) were downloaded from NCBI Genome Browser [54]. Evolutionary relationships of the above species were retrieved from TimeTree [55].

### Identification of putative MMPs

Sequences of the human MMPs were retrieved from UCSC genome browser [56]. MMPs from non-human primate species were identified by a combination of BLASTp and HMMER searches. By using BLASTp, searches were performed using an E-value cutoff of 1e-10 with 24 human MMP peptide sequences as queries [57]. For HMMER, hmmsearch (HMMER v3.3 package) was used to search for Pfam domain PF00413.23 as a profile with default settings [25, 58]. The searching results were combined and redundant sequences were removed. The identified genes were validated by searching the peptide sequences for the Hidden Markov Model profile of Pfam domain PF00413.23, the signature conserved domain of MMPs.

### Alignment and phylogenetic inference

Multiple sequence alignments of peptides were performed using MUSCLE v3.8.1551 [59]. Maximum likelihood phylogenetic trees were reconstructed with PhyML v3.3.3 package, with the confidence level of each branch estimated by using 1000 bootstrap replications and WAG model of amino acid evolution [60]. The phylogenetic trees were visualized with Figtree v1.4.4 (http://tree.bio.ed.ac.uk/software/figtree/). The primate MMP genes identified in this article were classified and designated based on the phylogenetic topology and Fanjul-Fenandex et al. [24].

### Evolution analysis of MMPs

Sequence divergence of each MMP clade was analyzed using codon substitution model implemented in CodeML under PAML 4.0 [61]. Two models, M7 and M8, of the distribution across amino acid sites were used, by which M7 assumes that ω is ß distributed along the sequence with a range limited by 0 and 1, while M8 assumes positive selection at some sites and the likelihood ratio test (LRT) of M8 against M7 provides a statistical test for positive selection. Sites of positive selection with P-value above 0.95 (ω>1) were retrieved. Nonsynonymous substitutions (*Ka*), synonymous substitutions (*Ks*) and *Ka/Ks* ratio between human and gorilla MMP orthologs were also calculated, which was implemented in codeML under PAML 4.0 [61].

### Identification of domains and signal peptides

Conserved domains of the peptide sequences were identified by using hmmsearch under HMMER v3.3 package against Pfam-A database with default trusted cutoffs [25, 58]. Signal peptides for the peptides were identified by querying the SignalP-5.0 server [62]. The location of domain and signal peptides were visualized by querying Gene Structure Display Server 2.0 [63].

### Gene structure characterization

Peptide molecular weights were calculated by using Bachem’s Peptide Calculator (https://www.bachem.com/knowledge-center/peptide-calculator/). Gene structure information was retrieved from GFF3 files. Gene length was calculated from the start to the end point of each gene, and CDS length was calculated by deducting intron lengths from gene length. While MMP-23s are classified in furin-activatable MMPs, they demonstrate very different characteristics than other MMPs, and thus were investigated separately.

### Genomic localization of MMP genes and co-synteny analysis

Localization of genes was retrieved from GFF3 files. The site of the start codon was considered as the gene localization of each gene. The length of each gene was calculated based on the genome sequences. The visualization of the genomics localization was performed by using MapChart 2.32 [64]. Co-synteny analysis was performed by using the MCScanX package [65].

### Gene expression analysis

Data of Illumina’s Human BodyMap 2.0 were downloaded from Expression Atlas at EBI (https://www.ebi.ac.uk/gxa/), and FPKM values of human MMPs were extracted. Longitudinal expression data for human testis and ovary were retrieved from GTEx Portal [32]. FPKM values of four non-human primates were extracted from non-human primate reference transcriptome resource (NHPRTR) [66]. Spearman’s correlation coefficient was calculated for each human gene pair and each MMP gene between primate species, and heatmaps were drawn using R package corrplot [67].

## Supplementary Material

Supplementary Table 1

Supplementary Table 2

Supplementary Table 3
